# Exploring the Multi-Layered Affordances of Composing and Performing Interactive Music with Responsive Technologies

**DOI:** 10.3389/fpsyg.2017.01701

**Published:** 2017-09-29

**Authors:** Anna Einarsson, Tom Ziemke

**Affiliations:** ^1^Department of Composition, Conducting and Music Theory, Royal College of Music in Stockholm, Stockholm, Sweden; ^2^Cognition and Interaction Lab, Human-Centered Systems Division, Department of Computer and Information Science, Linköping University, Linköping, Sweden; ^3^Interaction Lab, School of Informatics, University of Skövde, Skövde, Sweden

**Keywords:** affordances, cultural affordances, embodied activity, embodied cognition, composition, interactive music, responsive technology, situated activity

## Abstract

The question motivating the work presented here, starting from a view of music as embodied and situated activity, is how can we account for the complexity of interactive music performance situations. These are situations in which human performers interact with responsive technologies, such as sensor-driven technology or sound synthesis affected by analysis of the performed sound signal. This requires investigating in detail the underlying mechanisms, but also providing a more holistic approach that does not lose track of the complex whole constituted by the interactions and relationships of composers, performers, audience, technologies, etc. The concept of affordances has frequently been invoked in musical research, which has seen a “*bodily turn*” in recent years, similar to the development of the embodied cognition approach in the cognitive sciences. We therefore begin by broadly delineating its usage in the cognitive sciences in general, and in music research in particular. We argue that what is still missing in the discourse on musical affordances is an encompassing theoretical framework incorporating the sociocultural dimensions that are fundamental to the situatedness and embodiment of interactive music performance and composition. We further argue that the *cultural affordances* framework, proposed by Rietveld and Kiverstein (2014) and recently articulated further by Ramstead et al. (2016) in this journal, although not previously applied to music, constitutes a promising starting point. It captures and elucidates this complex web of relationships in terms of shared landscapes and individual fields of affordances. We illustrate this with examples foremost from the first author's artistic work as composer and performer of interactive music. This sheds new light on musical composition as a process of construction—and embodied mental simulation—of situations, guiding the performers' and audience's attention in shifting fields of affordances. More generally, we believe that the theoretical perspectives and concrete examples discussed in this paper help to elucidate how situations—and with them affordances—are dynamically constructed through the interactions of various mechanisms as people engage in embodied and situated activity.

## Introduction

Given that this paper deals with music, but is submitted to a cognitive science/psychology journal, we assume that the majority of readers are cognitive scientists, and only a minority of readers are familiar with music theory. The first question that comes to mind for the average cognitive scientist, who is to some degree familiar with Gibson's (1979/1986) notion of *affordances*, might be whether music really has affordances in the first place. After all, Gibson was mainly concerned with the sense of vision and the affordances of concrete physical objects. These were affordances conveyed by the optical array and perceived by agents being far from stationary, but moving about interacting with those objects, such as the *sit-ability* of chairs or the *grasp-ability* of cups. Nonetheless, hearing also deals with concrete objects, since sound carries information about a source. We are always in search for what is causing the sound, learning about environmental occurrences (Gaver, 1993; Windsor, 2000). As Jonas (1966/2001) writes, hearing is related to event and not to existence. Half a century later, Gibson's *ecological psychology* is still highly influential, not least in research on *embodied cognition* (e.g., Varela et al., 1991; Chemero, 2009; Shapiro, 2011), and, although widely debated, the concept of affordance is still very much used (e.g., Thill et al., 2013; Sakreida et al., 2016) and new conceptual frameworks are continuously being developed (e.g., Rietveld and Kiverstein, 2014; Ramstead et al., 2016; Davis and Chouinard, 2017).

In musical research, perhaps contrary to what one may assume, discussing the affordances of music is nothing new. It is in accord with the more over-arching *bodily turn* of musicology and related fields since the beginning of the twenty-first century (Pelinski, 2005). There is in fact a growing body of support for music as embodied and situated activity *Performing and interacting with musical instruments*, for example, is widely recognized as an embodied phenomenon (e.g., Leman, 2007; Windsor and De Bézenac, 2012). Furthermore, Clarke (2005), among others, has discussed the role of embodiment in the experience of music, particularly *listening*, and there also is support for an activation of the human mirror neuron system when *experiencing music* (Molnar-Szakacs and Overy, 2006). The concept of affordance has been used in music by a number of authors in recent years (e.g., Windsor, 1995, 2000; Clarke, 2005; Leman, 2007; Krueger, 2011, 2014; Menin and Schiavio, 2012; Windsor and De Bézenac, 2012; Einarsson, in press). It offers unique ways of describing the reciprocal relationship between performer/composer and musical structures, but also, as we will see, toward the performance situation as a whole, in all its complexity. Windsor and De Bézenac (2012), for example, have argued that “the concept of affordances helps to conceptualize the mutual relationships that exist between listeners and sounding objects and events, between performers and their instruments, and between musicians in a manner quite foreign to more cognitive structural approaches to music psychology” (2012, p. 103). This reciprocity being a topic of great research interest is emphasized also by Geeves and Sutton (2014). However, current interpretations of the concept of affordances in musical research vary very significantly among each other. Most of them also deviate significantly from the Gibsonian notion of affordances, which is not always acknowledged by the authors (as will be discussed in detail in the next section). As Davis and Chouinard (2017) state in their discussion of the general use of the affordance concept, the challenge for researchers is to delineate their usage of the concept and adopt this in ways displaying both relational, material and dynamic dimensions. We agree with them that the mechanisms of affordances operate within a situation, whose aspects interact and thus affect the efficacy of affordances, a notion highly applicable to music.

Over the last decade questions of *aliveness* and *embodiment*, in the light of advancements in technology (i.e., increased computer processing speed enabling interactivity between agents and computer system(s) to be staged and performed live in real time), have been a major concern in artistic fields such as *performance studies* (Broadhurst and Machon, 2011; Barrett and Bolt, 2013), *dance* (Kozel, 2007), and *music* (Emmerson, 2007; Peters et al., 2012). In the field of music, an interesting special case, in our opinion, is music whose composition and performance is aided by computer technology in real time (running time), i.e., *live electronic music*. We are particularly interested in interactive music utilizing responsive technologies, such as sensor-driven technology or, as the major focus in this article, computer sound synthesis affected by computer analysis of an acoustically performed sound signal. For example, features of a sung input (e.g. vibrato) are analyzed by the computer, and the subsequent electronic sounding (e.g., a chord) is dependent on the amount of vibrato. In musical works of this kind, a notion of interacting with “a disembodied other” (Emmerson, 2009) (i.e., computer technology), brings questions of embodiment and music to the heart of the discussion. We believe that the notion of affordances—in the broadened sense of *cultural affordances* discussed in this paper—can play a central role in such endeavors.

Hence, the aim of this paper is threefold: Firstly, to expand on the notion of affordances as it has been used in musical research previously, by clarifying the diversity of interpretations of the concept, but also the limitations of its present use. Secondly, to suggest an application of the notion of *cultural affordances*—originally proposed by Rietveld and Kiverstein (2014) and recently further elaborated by Ramstead et al. (2016) in this journal—to interactive music, where the performers, the audience, and the composer shape, experience, and perform music with and through individual yet overlapping, and dynamically varying, fields of affordances. This will be illustrated with examples from the first author's artistic work as composer and performer of *mixed works*, where a combination of acoustic sound sources (singers) and digital sound sources (responsive computer technologies) perform together live. Last, but not least, the focus of this paper is on musical performers' and composers' skill and embodied affective appraisal in dynamic relationship with the environment, situated inside a sociocultural practice. In our opinion, this contributes to bridging the conceptual gaps between the seemingly disembodied work of the composer, the concrete embodied activity of musical performers, their interaction with more or less “invisible” technologies, and the—according to some—highly abstract social and cultural practices that they are part of.

## On affordances

In order to contextualize the discussion, without any attempt to provide a complete historical account here^1^, we will recapitulate some relevant notions of the concept of affordances in psychology/cognitive science in general and elucidate its use in music research in particular.

### Affordances in the cognitive sciences

#### The reciprocity between organism and environment

Most of James J. Gibson's *ecological psychology* and his theory of perception were formulated in the late 1960s and−70s, i.e., long before embodiment had become a popular topic in the cognitive sciences. His work was a reaction against a mechanistic worldview and a move away from seeing cognitive processing as causation. First and foremost his work was concerned with visual perception, such as his influential theory of the visual field and the *optical array* (Gibson, 1979/1986).

Gibson introduced the notion of *affordances* for what he viewed as action opportunities for humans, or other animals, in their environment. In Gibson's original sense these have a peculiar ontological status: they are neither a property of the environment alone, nor a feature of the animal alone, but rather a property of both, i.e., emerging from the animal's interaction with its environment. In Gibson's own words:

*[…] an affordance is neither an objective property nor a subjective property; or it is both if you like. An affordance cuts across the dichotomy of subjective–objective and helps us to understand its inadequacy. It is equally a fact of the environment and a fact of behavior. It is both physical and psychical, yet neither. An affordance points both ways, to the environment and to the observer (Gibson*, *1979/1986**, p. 129)*.

Hence, a key aspect of affordances is that they are not just physical properties, but have to be considered relative to the animal. This reciprocity between organism and environment is fundamental to the Gibsonian notion of affordances. Affordances are specified by the pick-up of invariant information from the ambient light, the so-called *optical array*, whilst the animal—its body, legs, hands and mouth—is *coperceived* (Gibson, 1979/1986, p. 141). Thus, information does not equal affordances—information only points toward affordances. Furthermore, affordances, according to Gibson, are permanent and stable. They do not change relative to the organism's varying internal states, such as needs or motives. He writes: “Something that looks good today may look bad tomorrow but what it actually offers the observer will be the same” (Gibson, 1982, p. 410). This is, of course, not uncontroversial, because it means that, for example, a particular staircase is either—in principle—“climbable” for you or it is not, but its “climb-ability” does not vary over time just because some days you might be, for example, too tired or too drunk to actually climb it. So, to Gibson it is a reciprocal concept between organism and environment, but it is binary and relies on properties, which do not change according to changing needs. At this point, you might ask if affordances are opportunities for behavior, why do we not act on every possibility? What about social and cultural influences? And what about affordances not so readily available? These are issues we will come back to in the following.

As often is noted, Gibson's writings are sometimes ambiguous, some would say incomplete, and his theories have been a target of substantial criticism (e.g., Fodor and Pylyshyn, 1981). Nevertheless, his theories have undoubtedly spurred lots of interesting research and debate in the field. There are different interpretations and reformulations of Gibson's original theory, some of which have focussed more on the agent, some more on the object, and others have attempted to stay close to Gibson's original relational concept encompassing both agent and object.

#### From dispositional properties to relational abilities

Turvey, Shaw, and Mace took up the challenge of developing Gibson's ideas into a more philosophically sound and empirically tractable theory through a number of papers (e.g., Turvey et al., 1981). For them it was dispositional properties in the object and in the organism that enable action. They introduced the concept of *effectivities* (Shaw et al., 1982), complimentary to affordances, and intended to specify an animal's means for action, i.e., a combination of the function of its tissues and organs relative to the environment, to realize a specific affordance in a given situation. That means, the dispositional affordance and the effectivity complement one another. Hence, their theory relies on ecological laws, which are not universal but relate to a niche.

In particular this latter aspect has been one of the major criticisms formulated by Chemero (2006, 2009). Although he recognizes Turvey, Shaw, and Mace's contributions to the development of Gibson's ecological theory, his point is that they have turned the theory into having too little information available for direct perception, ruling out direct perception of *individuals* and perception of things partly determined by *convention*. “If information depends on laws,” he writes, “there is also no information about individual people available for perception. So although a human infant might have information available about humans, she has none about her mother” (Chemero, 2009). Moreover, ecological laws may structure the way that, for example, light is reflected off of an aluminium can, but according to Chemero they cannot account for instances where, for example, there has been a mix up in the factory between milk and soda, or someone has played a practical joke. Conventions build upon public agreement and are easily violated, he states.

Chemero's (2009) own take on modernizing Gibson, is—in a nutshell—to combine Gibson's theory with dynamic systems theory (also employing situational semantics in order to avoid natural laws and instead allowing for constraints connecting situations, which may be cultural or conventional). This is part of the formulation of what he refers to as *radical embodied cognitive science*. He argues that affordances are *relations* in a similar sense as one entity is taller than another. He also makes an important distinction between *feature* and *property*. While perceiving a feature is a matter of perceiving that *the situation as a whole has a certain feature*, perceiving a property, on the other hand, presupposes much more previous knowledge than perceiving features. Perceiving affordances, according to Chemero, is placing features. Secondly, Chemero argues, that instead of talking about an individual's capacities in terms of body scales, we should consider how an individual's ability is more relational. Dispositions never fail, but abilities may, thus allowing us to account for occasions when performance does not meet up with for example biological expectations (or where musical performances fail!). For example, one day somebody might simply be too tired to walk the steep stairs. Affordances and abilities, according to Chemero, causally interact and are causally dependent. That means, what Chemero refers to as *affordance 2.0* is a relation between the abilities of the individual and features of the environment.

#### Affordances as aspects of a sociocultural environment

Rietveld and Kiverstein (2014) propose a significantly broader application of affordances than Chemero. They emphasize how the exercise of abilities happens in a *context*, and that we as humans participate in *sociocultural practices*. Their two key concerns are: (1) the notion of a *form of life*, denoting human patterned behavior, i.e., “normative behaviours and customs of our communities” (ibid, p. 328), a concept borrowed from Wittgenstein, and (2) the influence of normativity on our engagement with affordances. Instead of features they prefer speaking of *aspects of an environment*, since “in the human case the material environment has been sculpted by our sociocultural practices into a sociomaterial environment (ibid, p. 335). Accordingly, they suggest the following definition: “Affordances are relations between aspects of a material environment and abilities available in a form of life” (ibid, p. 335). This is very much in line with Chemero's (2009) argument that “the situation as a whole supports (perhaps demands) a certain kind of action” (cf. Affordances in the Cognitive Sciences). In other words, this view enables us to consider the reciprocity between human and environment as conveyed by learned behaviors under the influence of social niches and conventions.

To Rietveld and Kiverstein, affordances are both relational and a resource (ibid, p. 327). They are relational in that they depend on the material environment and the abilities in the form of life. At the same time, they are resources in the way opportunities for action rely on how we create, for example, tools for our projects and concerns, and engage with changing aspects of the material situation. In their reading of Gibson, instead of affordances, they give primacy to the ecological niche for a kind of animal with a certain form of life. Accordingly, they introduce the notion of a *landscape of affordances*, meaning affordances available in an ecological *niche*. As Bruineberg and Rietveld (2014) put it: “In our human form of life, these are related to the whole spectrum of abilities available in our socio-cultural practices.” Furthermore, some affordances that “stand out more than others” to the individual (cf. Withagen et al., 2012), and which are relevant to a particular individual in a particular situation, are denoted as a *field of affordances* (Bruineberg and Rietveld, 2014). Sensitivity to a situation, to the landscape of affordances, is achieved through abilities or skills. These are in turn generally acquired through training and experience in sociocultural practices.

But, recapitulating the question in section From Dispositional Properties to Relational Abilities, how come we do not act on every affordance available in our field? According to Rietveld and Kiverstein, this is due to an agent's drive to achieve an *optimal grip* on the situation, a striving for improvement of the situation. The concept of optimal grip stems from philosopher Merleau-Ponty, intending to capture how living systems are always simultaneously “in a state of relative equilibrium and in a state of disequilibrium” (Merleau-Ponty in Kiverstein and Rietveld, 2015). Improving optimal grip entails a bodily action readiness: “In many real-life situations multiple states of action readiness interact in generating action tendencies and action” (ibid, p. 342). Ramstead et al. (2016) exemplify the concept of optimal grip nicely with the image of a boxer who orients toward the punching bag so as to afford a suitable variety of possible strikes. Optimal grip helps explaining the way some affordances in the field, through interaction with affective appraisal and attention, cause action readiness and become solicitations to the individual.

Lastly, Rietveld and Kiverstein (2014) also introduce the concept of *skilled intentionality*, i.e., “the individual's selective openness and responsiveness to a rich landscape of affordances” (Kiverstein and Rietveld, 2015) in overlapping cycles of action-perception. This ability is developed over the years as part of an increasing sensitivity to discriminate between situations. In other words, skilled intentionality is a tendency to act toward an optimal grip on a field of affordances (Bruineberg and Rietveld, 2014).

#### Cultural affordances

Ramstead et al. (2016), in their discussion of *cultural affordances*, building on Rietveld and Kiverstein (2014) and Kiverstein and Rietveld (2015), among others, have recently raised the question how culture and context interact with human biology to shape cognition, behavior, and experience. They distinguish between two kinds of cultural affordances: *natural affordances*, which are possibilities for action, dependent on agents' exploiting reliable correlations in its environment with its set of abilities (similar to Chemero's affordance 2.0) and *conventional affordances*, which depend on a shared set of expectations, norms and conventions. Important to note, there is a continuum of affordances between those that depend on reliable correlation (natural affordances) to those that depend on shared sets of expectations (conventional affordances). This view of affordances as gradual is also in accordance with Davis and Chouinard's (2017) recent characterization of affordances, as determined by and depending on a number of, in practice not easily discernable, situational cues. According to Ramstead et al. (2016), it is important to note that culture underpins *both* natural and conventional affordances. Herein also lies their definition of culture. In their own words: “Human biology is cultural biology; culture has roots in human biological capacities. The affordances with which human beings engage are cultural affordances.” Even more so, in their view, both kinds of affordances may be socially constructed. Hence, according to their theory, an affordance may be changed either by altering aspects of the material environment or the organism's form of life. Thus affordances may be shaped and vary in relation to enculturation, social influence, and skill, which is highly relevant to our discussion on musical practice.

Ramstead et al. (ibid) also adopt the concepts of a *landscape of affordances* (cf. previous section), which for them is relatively static and constituted by the totality of affordances available to a population in a given environment. They also use the notion of a *field of affordances*, which for them is the subset of “affordances in the landscape with which the organism, as an autonomous individual agent, dynamically copes and intelligently adapts,” i.e., “those affordances that actually engage the individual organism because they are salient at a given time, as a function of the interests, concerns, and states of the organism.” They argue, similar to Rietveld and Kiverstein (2014) that an organism does not encounter affordances one by one, but “as an ensemble of affordances, with which it dynamically copes and which it evaluates, often implicitly and automatically, for relevance.” These affordances are themselves entangled in various ways and appear as nested, depending on each other, hiding, enabling, or revealing other possibilities for action. Certain affordances, in this view, are also highly influenced by joint intentionality, social and cultural normativity and shared expectations (implicit and explicit), codetermining the landscape of affordances. The field of affordances is “experienced as “solicitations,” in that they solicit (further) affective appraisal and thereby prompt patterns of “action readiness,” that is, act as perceptual and affective prompts for the organism to act on the affordance.” This idea of affective appraisal causing readiness to act (cf. Lowe and Ziemke, 2011) is again highly relevant to a performer or composer's practice. Indeed, this means they depart from Gibson in a number of ways, including their argument that the individual experiences affordances as *solicitations*.

One of the assumptions the theoretical framework of conventional affordances rests upon is the dependence on shared expectations, or as they put it, how behavior is influenced by expectations about others' expectations. Accordingly, the presence of others affects the salience of affordances, due to human conventions. Also culturally shared expectations are embodied at various levels (brain networks, artifacts, constructed environments).

In effect, Ramstead et al. (2016) suggest a *predictive processing* model, emphasizing how the generative model does not need to entail semantic content. Generative models are embodied at different levels, may it be neurally in the brain or in terms of behavioral patterns. Here, attention plays a key role in guiding action perception, affecting the acquisition of culturally specific sets of expectation.

### Affordances in music research

How then do affordances in music work? Despite—or maybe due to—the extensive usage of the concept in music theory and related fields in recent years (e.g., Windsor, 1995, 2000; Clarke, 2005; Leman, 2007; Krueger, 2011, 2014; Menin and Schiavio, 2012; Windsor and De Bézenac, 2012; Einarsson, in press), interpretations and applications vary significantly. One thing most music scholars do seem to agree on though is that music *affords* movement (e.g., Clarke, 2005; Windsor and De Bézenac, 2012), although some focus mostly on aspects of synchronization or *entrainment* (e.g., Leman, 2007; Krueger, 2014), where entrainment, in Leman and Maes's (2014, p. 239) definition stands for “pre-reflective adaptation of human movement to music.”

To begin with, Clarke (2005), who primarily addresses the listener's point of view, accounts for culture as being a vital part of what we perceive. He writes on culture: “once a tradition or convention is established and is embodied in widespread and relatively permanent objects and practices, it becomes as much a part of the environment as any other feature” (ibid). In his view, music, carrying invariant structures, can reveal almost any source in a situation: instrument, medium, social functions in which they participate, emotional states, bodily actions, and spaces. Moreover, according to Clarke, affordances change in accordance with an organism's changing needs. He acknowledges that there are social constraints that make some affordances less likely, for instance a violin that affords burning, but does not elaborate on these aspects.

To Windsor and De Bézenac (2012), in line with Clarke, affordances are not fixed. Their view on culture follows Sanders (1997), entailing a stance according to which direct perception of affordances both may and should be applied in complex cultural contexts (Windsor and De Bézenac, 2012)—again, in line with Clarke. However, unlike Clarke, Windsor and Bézenac's approach resembles Reed's adaptation of Gibson (cf. Withagen et al., 2012), according to which affordances exert a selective pressure on the behavior of individuals. Windsor et al. also adopt from Shaw et al. (1982) the concept of *effectivities* (cf. section From Dispositional Properties to Relational Abilities).

Moreover, they acknowledge the relevance of other people and social/material context to human behavior, which they illustrate with the example of jazz musicians “going with” or “going against” what other musicians' actions afford. It is, however, not clear what the underlying mechanisms are. It is also somewhat contradictory how these affordances, on the one hand, can “determine the characteristics” of a particular music, while at the same time it is emphasized “that while the pianist's actions afford particular behaviours, they do not demand such behaviours” (ibid). Finally, a more controversial stance of theirs is that music affords semiotic acts, and the production of particular signs, for example through verbal or textual action (ibid, p. 114). All in all, Windsor and Bézenac make a substantial contribution to the discussion of different ways of applying direct perception and affordances to music, and do include music-making to a larger degree than Clarke. Still, we lack the full picture, and the concept remains far from being well defined.

The focus of Krueger (2014), another influential voice, on the other hand, is on emotion regulation. His view is one of *distributed cognition* (Hutchins, 1995), according to which music is for off-loading emotional responses. He equates affordances with the concept of *invites* (Withagen et al., 2012), but in a manner rather different from what Withagen et al. intended (ibid). He assigns a demand character to the concept of affordance, discussing them from a perspective of “the way that we often experience music as affectively irresistible” (Krueger, 2014, p. 2), and draws upon the notion of *entrainment* (see section Affordances in Music Research). Music, according to Kreuger, is part of a distributed system where “musical affordances provide resources and feedback that loop back onto us and in so doing, enhance the functional complexity of various motor, attentional, and regulative capacities responsible for generating and sustaining emotional experience” (ibid). Kreuger focuses on the listener's point of view, and although he is more detailed than Clarke (2005) or Windsor and De Bézenac (2012) regarding the theoretical underpinnings of this position—drawing upon, amongst other things, *the extended mind hypothesis* (Clark and Chalmers, 1998)—his focus is rather narrowly set on solicitations of different emotional experiences. Hence, his theory is difficult to apply to a performance situation as a sole theory. He only touches slightly upon any social dimension in terms of *affective synchrony*, albeit not particularly in relation to affordances, and culture is addressed only as a consequence of discussing the many contexts in which music can be utilized.

Menin and Schiavio (2012), finally, delimit, but also reinterpret the concept of affordances as dealing with *intentional* relationships between musical subjects and objects exclusively, a relationship grounded in how the motor possibilities of the subject's body can interact with the environment. Therefore they reject the idea of inferential relationships—such as, for example, a movie trailer “affording” going to the cinema—as being affordances. They draw a parallel to the work of Delalande (in ibid, p. 210) on children's exploratory behavior toward musical objects, concluding how embodiment (and the discovery of musical affordances as intentional acts) arises from sensory-motor modalities of interaction with an object. Thus their stance relies on relationships that have emerged during early childhood discoveries such as plunging, hitting and scratching. Accordingly, they propose “an embodied approach that radically diverges from the standard accounts, considering musical objects as entities constituted within the intentional motor-based relation that defines a musical context” (ibid, p. 211). It is not at all clear, however, how—and to what degree (if any)—they consider cultural or social aspects to influence or be part of the embodiment musical theory they describe.

### Where do we go from here?

**Figure 1:**
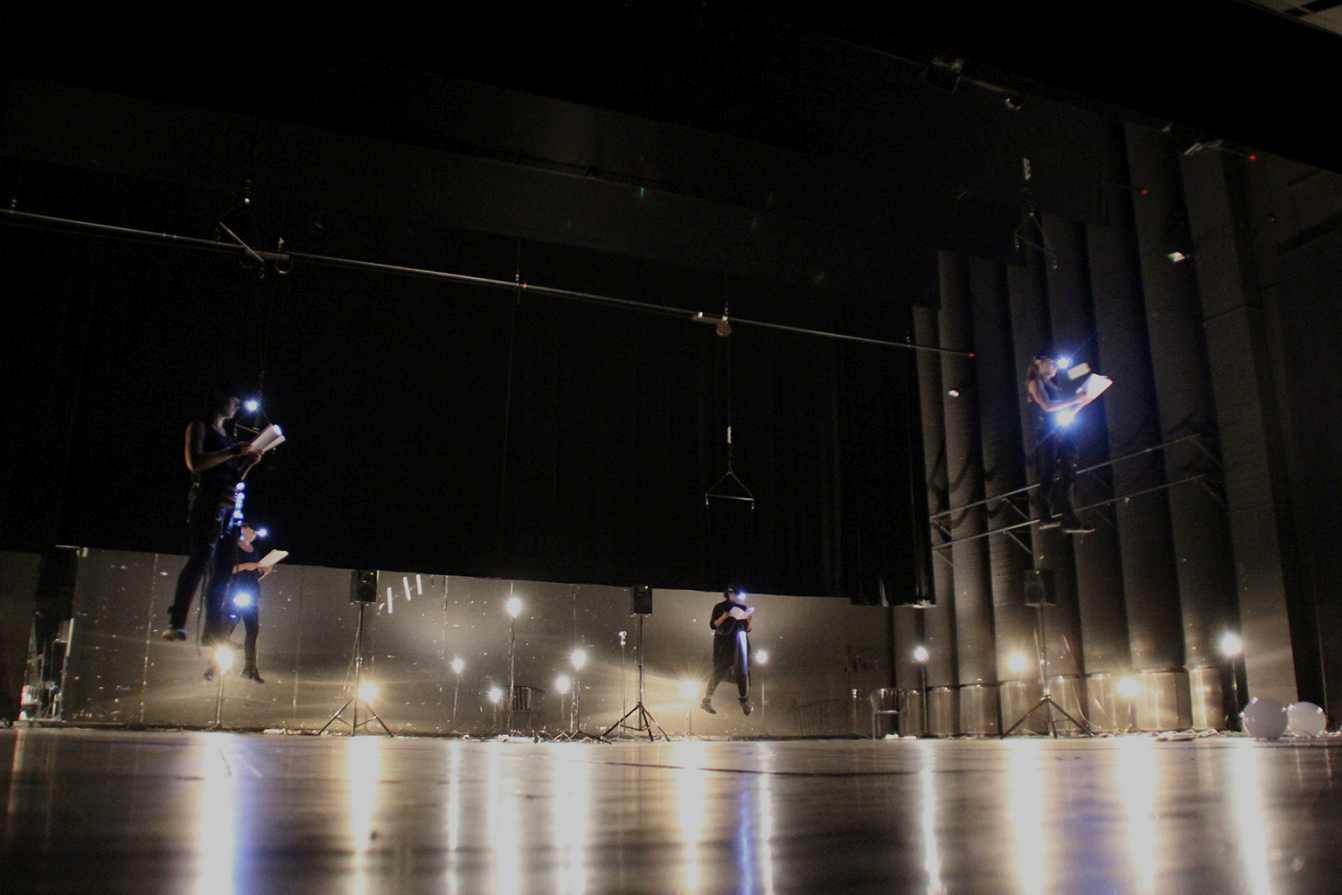
An illustration of the performance piece *Metamorphoses* (Einarsson, 2015a), a mixed work composed and co-performed by the first author (Einarsson, in press; see also https://vimeo.com/153345880 for a Vimeo video).

To summarize, in most music theorists' interpretations of affordances, cultural aspects are inevitably included, while the degree to which social aspects are incorporated varies to a large extent. What is still missing in the field of music research, in our opinion, is a more encompassing theoretical framework incorporating the sociocultural dimensions that are fundamental to the situatedness and embodiment of music performance, providing a detailed account of the underlying mechanisms, but also providing a more holistic approach that does not lose track of the complex whole constituted by the interaction of composers, performers, audience, technologies, etc. We believe that Ramstead et al.'s (2016) *cultural affordances* framework, as discussed in the previous subsection, although not previously applied to music, constitutes a promising starting point for capturing and elucidating this complex web of relationships. We will therefore in the next section illustrate this with examples foremost from the first author's artistic works as composer and performer of *mixed works*, where a combination of acoustic sound sources [singer(s)] and digital sound sources (responsive computer technologies) perform together live (cf. Figure 1).

## Affordances in interactive music

The mechanisms of affordances in music operate within a situation whose aspects interact and thus affect the efficacy of affordances. Hence, affordances and situation are inevitably intertwined. However, for the sake of analysis, we will attempt to address in the following different parameters as if they were separable. Importantly though, by discussing affordances in terms of aspects of a situation, as Rietveld and Kiverstein (2014) proposed, this enables us to address affordances as graded instead of binary, which is much more applicable to the reciprocal dynamics that are crucial to music performance in general, and interactive music in particular.

### The landscape and fields of affordances

What then constitutes the shared landscape of affordances in an interactive music work of which a performer is part? The landscape (cf. section Cultural Affordances) is the totality of available affordances in a niche, associated with a form of life, so for most cases it is the action possibilities offered by the audience, the concert space, the reciprocal relationship toward sounds generated by the computer technology and possibly other participating performers. As the framework suggests (cf. section Cultural Affordances), there are cultural affordances of both *natural* kind and *conventional* kind. An example of the former is a chair on stage that affords sitting, and in the latter case a microphone that affords singing into. In an interactive performance work, such as the first author's *Metamorphoses* (Einarsson, in press; cf. Figure 1), the situation holding the landscape is very complex; the music composition is realized only when the computer technology is interacted with, and stage directions are added to the performance, e.g., physical actions such as walking, sitting, standing, and singing elevated in the air in harnesses. Affordances appear, just as Rietveld and Kiverstein (2014) state, as nested and as an ensemble, where situation and affordances are inevitably intertwined.

Fields of affordances, on the other hand, (cf. section Cultural Affordances), are at the level of the individual. What will stand out for the individual performer, thus constitute their field of affordances, is dependent on the performer's concerns, needs and abilities. These are in turn under the influence of enculturation, patterned practices, directed attention and shared expectations. Altogether this will color the performer's detection of possibilities for action nested in the interplay with the computer, other performers, the audience, and the performance space. Again, there typically are cultural affordances of both natural kind and conventional kind. An example of the former is the act of turning toward a sound suddenly projected from a specific loudspeaker.

The latter, conventional affordances, may be exemplified with a musical structure containing short sampled sounds lasting for 20–100 ms or so, (i.e., granular synthesis) implemented at one location in the work *Metamorphoses*. The structure invites a sort of mimicking, which the score also devises. This electronic response is dependent on the length of the sung input (alongside additional parameters), and elicits a way of singing where space is left for the computer response. Following this, the character of the response impacts the improvisation toward becoming more fragmented and the denser the response gets, it brings about more pause on part of the singers. Thus there is a potential to reshaping the affordance gradually toward a background texture, increasing the likeliness of soliciting contrasting musical gesture like silence (Einarsson, in press). Singing itself is an interesting subcase, for singing words evoke emotions, and these in turn will impact appraisal of the affordance field.

In some instances this distinction between natural and conventional may be less clear-cut, which at the same time illustrates how natural and conventional affordances are poles on a continuum rather than two distinct categories. For example, an interesting study by Berg et al. (2016) reveals how a classically trained pianist adjusts his playing in relation to the room acoustics. The study was based in a modern concert hall where ceiling height could be altered, and there were also listeners present. The larger the concert hall the longer the reverberation time, and the slower the tempo the pianist performed at became. Interestingly, there was also a heightened focus on details in the interpretation when the reverberation was shorter. So, modifications to the material environment, and the impact this had on the sociocultural situation (as constituted by for example performance practice, the character of the music and listener's expectations), influenced the pianist's behavior.

### Striving for optimal grip

One challenge that arises in artistic practice is, in comparison to many other activities we as humans engage in, the goal, or optimum, is not very clearly defined. Perhaps the goal, to a performer or composer, can be put as ways of being and engaging with/in music. As T.S. Eliot famously stated: “You are the music, while the music lasts.” On the other hand, as Bruineberg and Rietveld (2014) write, “the skilled individual does not necessarily have an explicit goal in mind, but rather is solicited by the environment in such a way as to improve her grip on the situation.” Striving toward *optimal grip* is thus according to them equivalent to “having an action readiness for dealing adequately with an affordance” (ibid). Our suggested “goal” in terms of ways of being in music, is constrained by the demands of the situation, its physical, social and cultural prerequisites. One prerequisite may simply be the artistic work to be performed or composed. There may also be inner constraints derived from the motivations behind engaging in music, in particular emotionally laden ones.

The ways for improving grip, as a performer, may therefore be a tending toward having the full palate of artistic expression made available, in relation to the situational demands. The performer may optimize feedback monitoring, placement of equipment, positioning in relation to the audience and/or fellow musicians, controlling muscular tension/level of anxiety in order to perform at his or her best, minimize possible distractions, rehearse, acknowledge and adapt to present room acoustics—just to mention some. The performer may also learn new behaviors, cf. modifying affordances by changing the form of life. For example, in the work *PS. I will be home soon* by the first author (Einarsson and Friberg, 2015), performers reported that they had to find new listening strategies in order to achieve a satisfactory interaction with the computer. Many aspects applicable to the performer's situation may also be applied to the composer's situation. In addition, the composer can be said to have a goal set in terms of a directedness—let us call it an affective bearing toward which the artistic course for the work is set. An affective appraisal is always present when acting. So the *skilled intentionality* (cf. above), i.e., striving for optimal grip, in this case, speaking from the first author's experience, is reflected in having concrete tools readily available for composition (computer, instruments, synthesizers etc.), but also in terms of having access to the desired bodily state (as Damasio denotes it), pertaining feelings and cognitive processes in accordance with the idea for the work. The composer, similar to what composer Vaggione (2001) describes, attempts to using his/her own body as a template when shaping and listening to the work in progress, making use of embodied simulation in order to work with expectations and directing the attention of as well performers as audience as the work proceeds. Affect, attention and affordances interact to sculpt a field of affordances, as Ramstead et al. (2016) put it. These aspects of *skilled intentionality* may be seen as ways of unveiling *embodied expectations in the landscape of affordances* (i.e., shared expectations embodied in material culture, social niches and patterned cultural practices, enabling the landscape of affordances), by hands-on testing and experiencing sounds and computer responses when composing.

### Attention and joint attention

As Ramstead et al. (2016) point out, constructed human environments, which we suggest a musical work may be likened with, work with soliciting certain expectations and directing attention. Attention impacts the ways the performer engages with the field of affordance. How a performer is attentive is shaped over the course of development, as part of an enculturation, thus ways of relating to computer responses in an interactive piece of music is part of a larger picture, where preconceptions in terms of ways of being attentive are part of how the performer attends to the music. Since parameters for analysis and synthesis not seldom change dynamically throughout the piece, many affordances are highly dynamic.

Drawing upon interviews with singers from two different musical works, it is possible to compare a classically trained vocalists' conceptualisation of the computer (Einarsson and Friberg, 2015) with jazz vocalists' preconceptions of the computer (Einarsson, in press). These differences in sociocultural situation between singers identifying with different genres, i.e., different fields of affordances, show how waywardness in the relationship toward the computer may cause uncertainty in some singers, but the appraisal of uncertainty and subsequent course of action may vary very much due to what formal training (enculturation and skill) they have and what connotations the computer brings along (the object). Uncertainty was experienced as inherently negative by the classically trained vocalist, while to the jazz singers it was at the heart of the practice and to a large degree indispensible (Einarsson and Friberg, 2015; Einarsson, in press).

The singer's accounts in the work *Metamorphoses* (ibid) also reveal expectations, such as listening out for what is not already there, in other words, listening out for where the piece of music is heading trying to anticipate the computer's (re-) action, or trying to “un-listen” what some singers or computers are performing in order to execute difficult passages. This directly relates to the agent's selective engagement with the field of affordance, as modulated by directed attention.

According to Ramstead et al. (2016), joint and shared attention mark out some affordances as more salient, and this we suggest is part of how the composer works, i.e., by guiding the attention of both performers and audience. Particularly with interactive works, the first author's research brings forward performers' experiences of putting the relationship toward the computer on display for the audience or for fellow musicians (Einarsson, in press) in a “look what I found” sense. For example, the violinist in *PS. I will be home soon!* (Einarsson, 2012), performing in a motion-tracking system, described how she wanted to show the sounds to the audience. Through her path across the floor, where the motion detector tracked her movements, she achieved this display. Simply put, in one moment, the audience afforded the action of putting on display, and the electronic sounding afforded exploration and movement, yet these affordances can be assumed to interact, similar to what is suggested by Ramstead et al. (2016), which also would be interesting grounds for continuous study. This also applies to a mechanism only briefly touched upon by Ramstead et al (ibid), a description of how joint attention, usually only applied to dyadic relationships, may be projected to larger groups. The first author's research suggests that the musical work containing interactive technology may constitute one such case of expanded joint attention, where computer technology is part of the field of affordance holding an ensemble of nested affordances.

### Sociocultural dimensions

Recurring in this discussion, the musical performance situation is indeed a *sociocultural environment*, but as previously noted in section Affordances in Music Research, this is surprisingly often not addressed when discussing affordances in music. For instance, this entails that fellow musicians influence available affordances by directing attention to certain aspects of the landscape, making some behavioral responses more likely due to expectations based on formal training and experience than others.

Already Gibson spoke of information, of secondary knowledge, as a way of accessing some affordances, and by emphasizing similar sociocultural dimensions, as Rietveld and Kiverstein (2014) and later Ramstead et al. (2016) do, the theory makes much more sense in the field of music. For example, in an interactive musical work, for a performer to have some of the background information, such as knowing the composer's intentions with the relationships between materials, contributes to the sense of a whole and the discovery of affordances, i.e., how to choose between actions (Einarsson, in press).

One mechanism at work, affecting the fields of affordances for all parties involved (performers, audience, composer), is *sociocultural normativity*. This includes, but is by no means restricted to, (1) cultural artifacts such as the score, enculturation in terms of the singer's formal training, the ease with which certain actions are preferred over others—i.e., the ability of the performer, the participating institution (s), or (2) social influence such as the presence and proximity of the audience, the presence and proximity of other musicians, composer and technicians/staff, even social identity in terms of members of a social group not present at the moment. In interactive works it is apparent how emotions as well as culture and social relations are part of the interplay between performer and computer technology. Returning to the notion of experiencing waywardness in the relationship toward the computer, the situatedness, the enculturation and social influence, impact how this is experienced. With four singers in *Metamorphoses*, all having the same sort of “fickle playmate,” creates, according to the singers' accounts (Einarsson, in press), a sense of a shared handling of the situation (social influence).

One kind of computer response commented on by the singers performing *Metamorphoses* (ibid) was imitation, a driving force that enforces social liking (Leman, 2007). Engaging with certain responses offers a give and take of imitative gestures between singer and computer. Many of the affordances in the responsive work are thus nested, or of give-and-take character, and taking the musical lead in one direction opens up an array of action possibilities in the next step.

### The role of the composer

Given what has been discussed so far, the role of the composer is then to shape dynamical fields of affordances accounting for their possible interactions, based on a shared landscape of affordance [cf. subject position in film theory (see Clarke, 2005), shaping a shared frame of reference for interpretation, but here with an emphasis also on action—among other things]. Within this larger landscape of affordances and the musical performance situation with all its parties and multiple layers, there are clusters and overlaps: the singers' somewhat permeable and overlapping fields of affordances, and the listeners' fields of affordances. Considering this—consciously or unconsciously—is part of the composer's practice. Even when composing, we suggest composers create their field of affordances to operate within, relying on mechanisms of predictive processing and embodied simulation. Quiet inner listening brings about action cues, and extracts of musical passages or certain sounds projected over loudspeakers in the studio also suggest musical action in an embodied manner. Anticipating and forming relationships, as well as playing with expectations, is many times at the core of the composer's practice. This is in line with Ramstead et al.'s statement: “The everyday phenomenology of affordances is one of possibilities for action and their variations; in other words, of *expecting* certain nested action possibilities and prescriptions for action” (Ramstead et al., 2016, p. 13, our emphasis).

An interesting example of working with the field of affordances is the audiovisual performance work One *piece of a shared space* (Einarsson, 2015b), where sung vowel sounds had an impact on the localisation of sound in the concert space (i.e., spatialization). The singer experienced the relationship toward the live electronics as quite ephemeral, and looking through the lens of the cultural affordances framework some interesting issues arise. A response in the domain of location does not first and foremost solicit an action of vocalizing. Rather the suggested action is to turn toward the sound, to approach and examine (a natural affordance). The concert space where this particular piece was rehearsed did not allow for very much movement, thus restricting the field, but when this kind of action was added as a kind of stage direction, to turn toward the sound (a guidance in the field of affordances), it did become more meaningful to watch and also made more sense to the performer. As a continuation, one could hypothesize that this kind of affordance would be better highlighted in an environment allowing for more exploration (changing the “form of life” by manipulating the environment, and/or behavior), or, a situation where the system was also susceptible for movement, i.e., the performer's movement was also taken into account for analyses, in addition to the sung input (changing the form of life by manipulating the material).

Hence, pertinent to our discussion of music performance is a dynamic between shared landscape and individual fields of affordances, and we suggest that considering this dynamic is at the heart of the music composer's practice. We are, however, not saying that compositional practice is devoid of rationalizations or structured approaches, but rather—following Damasio (1994), Johnson (2007), and Ziemke (2016)—that embodiment is fundamental to every aspect of human life and meaning-making.

## Discussion and conclusion

One of the driving forces behind this research has been the question how we can begin to account for the complexity of interactive music performance situations and analyze details without losing track of the whole. We have argued that what is still missing in the discourse on musical affordances is an encompassing theoretical framework incorporating the sociocultural dimensions that are fundamental to the situatedness and embodiment of interactive music performance. This would be facilitating a detailed account of the underlying mechanisms, but also providing a more holistic approach that does not lose track of the complex whole constituted by the interaction of composers, performers, audience, technologies, etc. We believe that Ramstead et al.'s *cultural affordances* framework, drawing upon the work by Rietveld and Kiverstein (2014), although not previously applied to music, constitutes a promising starting point for capturing and elucidating this complex web of relationships. Furthermore, by providing insights into the underlying mechanisms, it also facilitates new ways of considering the process toward new musical works as well as the performance situation as such. We hope to have illustrated this in this paper, at least to some degree, with examples from the first author's artistic work as composer and performer of *mixed works*, where a combination of acoustic sound sources (singers) and digital sound sources (responsive computer technologies) perform together live.

To begin with, Ramstead et al. (2016) put forward, echoing Rietveld and Kiverstein (2014), that an ecological niche equals a landscape of affordances. “The total ensemble of available affordances for a population in a given environment. This landscape corresponds to what evolutionary theorists in biology and anthropology call a ‘niche”’ (Ramstead et al., 2016, p. 3). We then learn how a niche: “[…] in the case of humans, the social world [is]—associated with (and partly constituted by) a form of life” (ibid, p. 5). We also learn that: “Different human communities, societies, and cultures, with sometimes strikingly different styles of engagement with the material and social world, constitute different forms of life.”

Hence, the consequence of what they are saying is, different forms of life entail different landscapes of affordances. Furthermore they describe how there is also a strong influence on available affordances in a niche from “local ontologies,” i.e., collective expectations, installed through specific ways of doing joint activities in domain-specific material-discursive environments (patterned practices). They write: “[…] these ontologies codetermine the exact affordances that are available in a given niche, for they prescribe certain ways of being, thinking perceiving and acting in context that are situationally appropriate” (ibid, p 14). So, local ontologies also influence affordances available in a niche, i.e., the landscape.

In our analysis, we have seen the need for a way to describe these arenas of a landscape of affordances where local ontologies derived from social niches and cultural practices have shaped a community as part of a landscape. Reading Ramstead et al. (2016) closely, they also seem to be grasping for this level of analysis:

“*Our claim here is that cultural affordances (especially conventional ones) form a coordinated affordance landscape, which is enabled by sets of embodied expectations that are shared by a given community or culture. Social niches and cultural practices generally involve not isolated, individual affordances or expectations but local landscapes that give rise to and depend on shared expectations. We submit that these shared expectations—implemented in the predictive hierarchies, embodied in material culture, and enacted in patterned practices—contribute to the constitution of the landscape of affordances that characterizes a given community or culture” (Ibid, p.14 our emphasis)*.

Kiverstein and Rietveld (2015) write: “The human landscape of affordances is one that is tightly interwoven with both material aspects and social and cultural practices *local to different regions of this landscape*” (ibid, p. 712 our emphasis).

We interpret this as a common reaching for an intermediate level between landscape and field, a “local landscape” in the words of Ramstead et al, or a “region of the landscape” in the words of Kiverstein and Rietveld (2015). In a similar vein, Kiverstein and Rietveld (ibid) touch upon how a landscape of affordances relies on possibilities for action available in a particular form of life, because of the patterned and coordinated activities in which members of this form of life are able to partake in. We see a need for this level constituted by clustered fields, *an arena of affordances*, for example when discussing performers identifying with different genres, stemming from different sociocultural background, i.e., different formal training, different repertoire knowledge, different ideals and expectations, and familiarity with different institutions and patterned practices associated with these. These differences are distinct and relatively stable, although not as distinct we would claim as to call them different forms of life, i.e., different landscapes. We therefore advocate an addition to the framework in terms of an intermediate level, an arena of affordances, meaning clustered fields of affordances determined by shared local ontologies and social and cultural practices, as part of the landscape of affordances.

Hence, what this paper contributes to the understanding of music as embodied and situated activity, we believe, is the presentation and illustration of a theoretical framework centered on affordances, yet a broader notion of affordances than previously discussed in the musical context. We argue that this is more suitable for capturing the social and cultural aspects that are central to musical performances, while also not losing track of their embodied nature. In our opinion, the crucial departure from the original Gibsonian notion of affordances, and many later variations and interpretations thereof, lies in the position that it is the *situation as whole that has affordances*. This also sheds new light, as discussed in detail in the previous section, on musical composition as a process of construction—and embodied mental simulation—of situations, guiding the performers' and audience's attention in shifting fields of affordances.

Finally, what this paper contributes to the research topic “*Beyond Embodied Cognition*”, is an illustration—using the case of interactive music—of how seemingly highly abstract, disembodied and unsituated activities, such as the composition of musical works, can in fact be strongly grounded in concrete embodied and situated activity. Hence, the contribution to the cognitive sciences in general, beyond the specific application to interactive music, lies in the formulation, discussion, and illustration of a significantly broader notion of affordances as *aspects of situations*, building on the recent work of Rietveld and Kiverstein (2014) and later Ramstead et al. (2016) on cultural affordances. The distinction between a population's relatively static landscape of affordances and individuals' dynamically varying fields of affordances, is also in line with recent work on the neural and cognitive mechanisms underlying affordances, which indicates that affordance perception is less direct, more context- and goal-dependent than Gibson thought 40–50 years ago (Thill et al., 2013), and that there are separate brain pathways for stable and variable affordances (Sakreida et al., 2016). The question of what exactly constitutes a situation is of course not trivial—that discussion goes back to at least Dewey (1938) and Russell (1939), and is beyond the scope of this paper. However, we believe that the theoretical perspectives and concrete examples discussed in this paper help to elucidate how situations—and with them affordances—are dynamically constructed through the interactions of biological, contextual, social, and cultural mechanisms as embodied and situated activity unfolds.

## Author contributions

The work reported here is part of AE's doctoral research, for which TZ has been the main supervisor. Accordingly, most of the text has been written by AE, and much of the material comes from her artistic work as well as her doctoral dissertation. TZ has contributed to framing the discussion from a cognitive science perspective.

### Conflict of interest statement

The authors declare that the research was conducted in the absence of any commercial or financial relationships that could be construed as a potential conflict of interest.

## Footnotes

^1^More complete historical accounts of the notion of affordances have recently been provided by, for example, Dotov et al. (2012) and Osiurak et al. (2017).
